# S100A8 Promotes Inflammation *via* Toll-Like Receptor 4 After Experimental Traumatic Brain Injury

**DOI:** 10.3389/fnins.2020.616559

**Published:** 2021-02-03

**Authors:** Guo-Yuan He, Chen-Hui Zhao, De-Gang Wu, Hao Cheng, Le-An Sun, De-Long Zhang, Xin-Jie Yang, Xi-Ran Fan, Guang-Fu Di, Xiao-Chun Jiang

**Affiliations:** ^1^Department of Neurosurgery, Yijishan Hospital, Wannan Medical College, Wuhu, China; ^2^Department of ICU, Tongling Clinical College of Anhui Medical University, Tongling, China

**Keywords:** S100A8, macrophage-related protein 8, inflammation, toll-like receptor 4, traumatic brain injury

## Abstract

**Introduction:**

S100 calcium-binding protein A8 (S100A8) is also known as macrophage-related protein 8, which is involved in various pathological processes in the central nervous system post-traumatic brain injury (TBI), and plays a critical role in inducing inflammatory cytokines. Accumulating evidences have indicated that toll-like receptor 4 (TLR4) is considered to be involved in inflammatory responses post TBI. The present study was designed to analyze the hypothesis that S100A8 is the key molecule that induces inflammation *via* TLR4 in TBI.

**Methods:**

The weight-drop TBI model was used and randomly implemented on mice that were categorized into six groups: Sham, NS, S100A8, S100A8+TAK-242, TBI, and TBI+TAK-242 groups. In the S100A8+TAK-242 and TBI+TAK-242 groups, at half an hour prior to the intracerebroventricular administration of S100A8 or TBI, mice were intraperitoneally treated with TAK-242 that acts as a selective antagonist and inhibitor of TLR4. Furthermore, the protein recombinant of S100A8 was injected into the lateral ventricle of the brain of mice in the S100A8 and S100A8+TAK-242 groups. Sterile normal saline was injected into the lateral ventricle in the NS group. To evaluate the association between S100A8 and TLR4, Western blot, immunofluorescence, enzyme-linked immunosorbent assay (ELISA), and Nissl staining were employed. Simultaneously, the neurological score and brain water content were assessed. In the *in vitro* analysis, BV-2 microglial cells were stimulated with lipopolysaccharide LPS or S100A8 recombinant protein, with or without TAK-242. The expression of the related proteins was subsequently detected by Western blot or enzyme-linked immunosorbent assay.

**Results:**

The levels of S100A8 protein and pro-inflammatory cytokines were significantly elevated after TBI. There was a reduction in the neurological scores of non-TBI animals with remarkable severe brain edema after the intracerebroventricular administration of S100A8. Furthermore, the TLR4, p-p65, and myeloid differentiation factor 88 (MyD88) levels were elevated after the administration of S100A8 or TBI, which could be restored by TAK-242. Meanwhile, in the *in vitro* analysis, due to the stimulation of S100A8 or LPS, there was an upregulation of p-p65 and MyD88, which could also be suppressed by TAK-242.

**Conclusion:**

The present study demonstrated that the TLR4-MyD88 pathway was activated by S100A8, which is essential for the development of inflammation in the brain after TBI.

## Introduction

Traumatic brain injury (TBI) is a serious public health problem due to the increase in the number of patients and disease severity (Maas et al., 2017). To date, more than 50 million people are affected by TBI every year, and it has been estimated that about half of the world’s population will have one or more TBIs over their lifetime (Maas et al., 2017). This has become a major cause of death and disability in affected people. Furthermore, TBI costs the global economy approximately $US 400 billion annually (Maas et al., 2017). In addition, TBI is associated with long-term risks, such as cognitive impairment, dementia (Reynolds et al., 2012; Li et al., 2016), stroke (Liao et al., 2014), parkinsonism (Jafari et al., 2013; Gardner et al., 2015; Crane et al., 2016), and increase in long-term mortality (McMillan et al., 2011; Stocchetti and Zanier, 2016). Various biochemical disturbances occur in the brain when there is TBI. The characteristic features of affected TBI individuals are white matter degradation, neuronal loss, protein misfolding, persistent neuroinflammation, and alterations in neurotransmitter systems (Stocchetti and Zanier, 2016). As soon as a TBI event occurs, an inflammatory response, such as the local cerebral production of cytokines and chemokines, endothelial activation, microglial activation, and the migration of systemic neutrophils, lymphocytes, and monocytes, rush toward the injured brain (Stocchetti and Zanier, 2016).

Furthermore, soon after the primary brain injury damage, evidence of a few pathological changes occur, such as cerebral blood flow (hypo- and hyper-perfusion), impairment of the cerebrovascular autoregulation, cerebral metabolic dysfunction, and inadequate cerebral oxygenation (Werner and Engelhard, 2007). Moreover, glutamate excitotoxicity cell damage, oxidative stress, increased vascular permeability, disturbance of ionic homeostasis, and inflammation in the brain may lead to apoptotic and necrotic cell death (Werner and Engelhard, 2007; Bell et al., 2009; Cornelius et al., 2013). This further leads to the secondary brain injury, in which the progression of tissue damage correlates to the direct release of neurotoxic mediators, or indirect release of nitric oxide (NO) and cytokines (Werner and Engelhard, 2007). The additional release of vasoconstrictors (prostaglandins and leukotrienes), the obliteration of microvasculature through the adhesion of leucocytes and platelets, the blood–brain barrier lesion, and the edema formation further reduce the tissue perfusion, and consequently aggravate the secondary brain damage (Werner and Engelhard, 2007).

When TBI occurs, it initiates the primary insult, and alters the neurochemical, cellular, and metabolic activity, which progresses into a secondary injury, and becomes a gradual process (Corrigan et al., 2016). Among these, inflammation plays a critical role in the brain damage after TBI, which is an important contributor to the neurological deficit (Lucas et al., 2006). At the same time, the activated microglial after TBI stimulates the NO that increases the permeability in the capillaries, and causes brain edema, blood–brain barrier dysfunction, and enhanced neuronal apoptosis (Therajaran et al., 2020). However, still there is no effective treatment available for TBI.

It is known that immune cells, such as neutrophils and monocytes, are considered as the first line of defense mechanism, which acts at the site of inflammation during an infection or sterile injury (Pruenster et al., 2016). Furthermore, these cells possess a large amount of S100A8, which is a heterodimeric protein present in the cytoplasm (Pruenster et al., 2016). S100A8 belongs to the Ca^2+^ sensor proteins that contain the specific Ca^2+^-binding motif (helix–loop–helix, called EF-hand). Moreover, the EF-hand has a high Ca^2+^ binding affinity toward a specific feature of the helix–loop–helix motif with charged amino acid residues (Wang et al., 2018). S100A8 has been successfully tested in pre-clinical imaging studies to localize at the site of infection or sterile injury (Pruenster et al., 2016). Finally, a recent evidence on the use of small molecule inhibitors for S100A8 also suggested that blocking S100A8 activity exerts beneficial effects on disease activity in animal models of autoimmune diseases, including multiple sclerosis, systemic lupus erythematosus, rheumatoid arthritis, and inflammatory bowel disease (Pruenster et al., 2016). Hence, S100A8 has gained importance, since it acts as a critical modulatory of inflammatory responses as soon as it is released from the neutrophils and monocytes (Pruenster et al., 2016). However, S100A8 has a significant potential to play multiple roles, such as intracellular S100A8 complexes that exhibit the function of amino acid metabolism, confer protection against pathogens, and cytoskeleton modulation (Wang et al., 2018). Furthermore, extracellular S100A8 participates in inducing the secretion of multiple cytokines and leukocyte recruitment (Wang et al., 2018). S100A8 is the main active component of the S100A8 complex (Vogl et al., 2007). Hence, the investigators mainly focused on the role of S100A8 in inducing TBI.

S100A8 and S100A9 (which are known as MRP8 and MRP14) are also known as damage-associated molecular pattern molecules that serve as warning signals in the inflammatory process (Ma et al., 2017). S100A8 is involved in the pathology of various diseases and injuries in the central nervous system (CNS), including TBI (Ziegler et al., 2009; Zlokovic, 2011). However, the downstream signaling pathway of the S100A8 induced after TBI remains unclear.

Increasing data has revealed that Toll-like receptors (TLRs) play an important role in innate immunity and inflammatory responses. The expression of TLR1–9 could be observed in microglia and macrophage-like glial cells in the brain (Olson and Miller, 2004). Among these, TLR4 can be activated by lipopolysaccharide (LPS), and is extensively expressed in the brain. It was reported in a previous study that in endotoxin-induced shock, S100A8 could stimulate TLR4 (Vogl et al., 2007). TLR4 was reported to be an essential receptor for the inflammatory responses that occur in the brain after TBI (Chen et al., 2009). The adapter protein myeloid differentiation factor 88 (MyD88) mediates the TLR4 signaling pathway, and subsequently activates the transcription factor nuclear factor-kappa B (NF-κB), in which numerous pro-inflammatory cytokines are produced such as IL-1β and TNF-α (Shi et al., 2013). Nevertheless, the relationship between S100A8 and the TLR4-MyD88 signaling pathway in response to TBI remains unknown.

Hence, the present study aimed to determine whether S100A8 promotes the expression of pro-inflammatory cytokines by activating the MyD88/NF-κB signaling pathway, and whether this is associated with TLR 4 in the brain after TBI.

## Materials and Methods

### Animal Preparation

In the preset experimental analysis, all procedures that included animals were ethically approved by the Animal Care and Use Committee of Jinling Hospital. Adult male, Institute of Cancer Research (ICR) mice (28–32 g) were purchased from the Animal Center of Qinglongshan (Nanjing, China). The gender difference had an effect on the neuroinflammation (Do Rego et al., 2009; Khaksari et al., 2017). In order to avoid this difference, the experiment was conducted only on males (Hopp et al., 2017; Liu et al., 2018). These mice were housed in cages, with a constant room temperature (25 ± 2°C) and air humidity (50 ± 10%). Furthermore, these mice were allowed to have free access to food and water, and undergo the light/dark cycle for 12 h.

### Experimental Designs

The present study was categorized into two parts: *in vivo* experiments and *in vitro* experiments.

#### *In vivo* Experiments

Initially, the cortical expression of S100A8 was assessed after TBI. For the experimental part, 21 adult, male ICR mice, weighing approximately 28–32 g, were selected and randomly divided into seven groups: Sham group (*n* = 3/group) and TBI groups (3, 6, 9, 12, 24, and 72 h) (*n* = 3/group). Sham mice were euthanized at 24 h, and the skin incision was performed only during the surgery. Furthermore, TBI mice were euthanized at the indicated time-point after TBI. Then, after euthanizing mice in each group, cerebral cortex tissues around the lesion site were collected and estimated by Western blot analysis. In addition, six other mice were added into the Sham (*n* = 3) and TBI (*n* = 3) groups, which were euthanized at exactly 24 h after the TBI, to collect all the brains for performing the immunofluorescence staining.

Furthermore, as the experiment proceeded, one-time point (24 h) was selected for the experiments, to perform this according to the time-course of the S100A8 expression. Afterward, 36 male mice were randomly divided into six groups: (1) sham group, (2) NS group, (3) TBI group, (4) TBI+TAK-242 group, (5) S100A8 group, and (6) S100A8+TAK-242 group for the Western blot, and 60 male mice were randomly divided into five groups: (1) sham group, (2) TBI group, (3) TBI+TAK-242 group, (4) S100A8 group, and (5) S100A8+TAK-242 group for Nissl staining, enzyme-linked immunosorbent assay (ELISA), and wet/dry method. For mice in the sham group, the skin incision was performed only during the surgery, and these mice were subsequently euthanized accurately at 24 h. Mice in the TBI group were euthanized, as mentioned below. TAK-242 (MCE; Monmouth Junction, NJ, United States) was intraperitoneally administered (0.3 mg/kg) to mice in the TBI+TAK-242 and S100A8+TAK-242 groups at 0.5 h prior to the intracerebroventricular administration of S100A8 or TBI. For mice in the S100A8 and S100A8+TAK-242 groups, S100A8 recombinant protein (3 μg) at a volume of 5 μl was injected into the lateral ventricle of the brain, according to a previous report (Yamada et al., 2011). However, for mice in the NS group, sterile normal saline (5 μl) was injected into the left lateral ventricle. Briefly, the coordinates for the intracerebroventricular injection were 1.8 mm lateral from the midsagittal suture, 0.7 mm posterior to the bregma, and -2.5 mm from the flat skull surface. The intracerebroventricular injection was performed at 30 min after the TAK-242 was intraperitoneally administered to mice.

To perform the immunological assays, the brain samples were collected for the Western blot (*n* = 6) and ELISA (*n* = 3). Nissl staining (*n* = 3, for each group) was also performed. In addition to the above techniques, the brain water content was measured by using the wet/dry method (*n* = 6), as described below. The neurological deficits (*n* = 6) were evaluated.

#### *In vitro* Experiments

BV-2 cells were cultured in six-well plates and were randomly divided into five groups: (1) sham group, (2) S100A8 group, (3) S100A8+TAK-242 group, (4) LPS group, and (5) LPS+TAK-242 group. Then, cells were collected for Western blot analysis (*n* = 3), and the cell-free supernatants were collected for ELISA (*n* = 3).

### Traumatic Brain Injury Model in Mice

In the present study, in order to perform the weight-drop model technique, the investigators followed the procedure applied by Flierl et al. (2009). Briefly, after the mice were etherized, these were placed onto a stereotaxic frame under the weight-drop device. To expose the skull, a 1.5-cm midline longitudinal scalp incision was performed on the head of mice. In order to achieve the target area, at 1.5 mm lateral to the midline on the mid-coronal plane, an impact was made using an object that weighed 200 g from a height of 3 cm onto the left lateral skull. Then, the investigators used 0.9% normal saline solution to transcardially perfuse the mice after these were deeply anesthetized *via* inhalation of isoflurane. Finally, the brain was collected for the other subsequent analyses.

### Neurological Scoring

The modified Garcia method was used to calculate the neurological score at 24 h after TBI by two investigators who were blinded of the grouping (Garcia et al., 1995). The modified Garcia method consisted of six tests, which were categorized, as follows: spontaneous activity (in the cage for 5 min), symmetry in the movement of the four limbs, symmetry of the forelimbs (outstretching while held by the tail), climbing wall of wire cage, reaction to touch on either side of the trunk, and response to vibrissae stimulation. The scores ranged within 3–18 (Table 1).

**TABLE 1:** Neurological evaluation in sham, S100A8, S100A8+TAK-242, TBI, and TBI+TAK-242 groups.

Test	Score	Score	Score	Score
				
	0	1	2	3
Spontaneous activity (in cage for 5 min)	No movement	Barely moves	Moves but does not approach at least three sides of cage	Moves and approaches at least three sides of cage
Symmetry in the movement of four limbs	Left side: no movement	Left side: slight movement	Left side: moves slowly	Both sides: move symmetrically
Climbing wall of wire cage		Fails to climb	Left side is weak	Normal climbing
Reaction to touch on either side of trunk		No response on left side	Weak response on left side	Symmetrical response
Response to vibrissae touch		No response on left side	Weak response on left side	Symmetrical response

### Brain Water Content

The brain water content was measured using the dry–wet weight method (Yabluchanskiy et al., 2012), in which the anesthetized mice’s brain was removed. Then, the right cerebral hemispheres, brain stem, cerebellum, and the remaining left cerebral hemispheres were removed and collected, and the wet weight (WW) was recorded. Afterward, the dry weight (DW) was obtained after the left hemispheres were dried at 72°C for 72 h. The results of brain water content were calculated using the formula: (WW - DW)/WW × 100%.

### BV-2 Microglial Cell Culture

To obtain the BV-2 microglial cells, these were purchased from Cobioer Biosciences Co., Ltd. (Nanjing, China), and cultured in Dulbecco’s modified Eagle medium (DMEM) (Sigma-Aldrich, St. Louis, MO, United States), which contained 5% of fetal bovine serum (FBS; Gibco, Grand Island, NY, United States) and 1% of penicillin/streptomycin (pen/strep; Gibco, Grand Island, NY, United States). Then, at a concentration of 10 nM, TAK-242 was added into the cultured cells an hour before the stimulation of S100A8 protein or LPS. These cells were treated with the S100A8 recombinant protein at a final concentration of 0.5 μl/ml, and LPS (Lipopolysaccharide, Sigma-Aldrich, St. Louis, MO, United States) was added to stimulate the BV-2 cell line at a final concentration of 0.1 μg/ml, according to a previous study (Henn et al., 2009).

### Western Blot Analysis

For the *in vivo* study, brain tissues located under the injured site were collected, and the protein was extracted using a Total Protein Extraction Kit (Beyotime, Nantong, China), according to manufacturer’s instructions. Then, the BCA Protein Assay Kit (Beyotime, Nantong, China) was used to determine the protein concentrations. Equal amounts of protein were gradually subjected to 10–12% of sodium dodecyl sulfate-polyacrylamide gel electrophoresis (SDS-PAGE), and transferred onto polyvinylidene difluoride (PVDF) membranes. Then, these membranes were blocked with 5% freshly prepared skim milk for 1–2 h and incubated overnight at 4°C with the following primary antibodies: S100A8 (1:1,000; Abcam, Cambridge, MA, United States), TLR4 (1:200; Santa Cruz Biotechnology Inc., Santa Cruz, CA, United States), MyD88 (1:500; Abcam, Cambridge, MA, United States), p-p65 (1:200; Santa Cruz Biotechnology Inc., Santa Cruz, CA, United States), and β-actin (1:5,000; Bioworld Technology, Bloomington, MN, United States). For the final step of the experiment, horseradish peroxidase (HRP)-linked secondary antibodies were used to incubate the membranes for 1 h. The blotted protein bands were visualized using an enhanced chemiluminescence (ECL) kit (EMD Millipore, Billerica, MA, United States), and were recorded by the Tanon 5500 Chemiluminescence Imaging system (Tanon Science and Technology Co., Ltd., Shanghai, China). The band density was quantified using ImageJ software (NIH, Bethesda, MA, United States), with normalization to β-actin.

Similarly, Western blot analysis was performed in the cultured BV2 cells, in which cells were lysed in lysis buffer that contained phosphatase inhibitor cocktail 2 (Sigma-Aldrich, St. Louis, MO, United States). Then, the protein concentrations were quantified using a BCA protein kit. Afterward, the samples (50 μg of protein per lane) were loaded on 12% SDS-PAGE gels. The following steps were consistent with the *in vivo* experiments.

### Enzyme-Linked Immunosorbent Assay

For the *in vivo* study, in order to quantify the release of cytokines from the brain after the TBI (*n* = 3/group), mice were sacrificed at 24th hour, these brain tissues were lysed with RIPA (Thermo Fisher Scientific, United States) with protease inhibitor (Roche, Switzerland), and homogenized thoroughly by sonication. The brain homogenates were centrifuged at 5,000 *g* for 5 min at 4°C. Cytokines, such as interleukin (IL)-1β and tumor necrosis factor-α (TNF-α), were quantified after obtaining the supernatant from a commercially available ELISA kit (both purchased from Multisciences, Hangzhou, China). All procedures were performed according to manufacturer’s protocols.

For the *in vitro* study, to perform the IL-1β and TNF-α assay using the ELISA kit, the samples were centrifuged at 1,000 *g* for 20 min at 4°C, and the cell-free supernatant was collected after centrifugation.

### Immunofluorescence Staining

Zhou et al. (2015) described the procedure of immunofluorescence staining, and this was performed to clearly visualize the images. Prior to this, the brain sections were incubated overnight at 4°C with rabbit anti-S100A8 antibody (1:200; Abcam, Cambridge, MA, United States). After washing with phosphate-buffered saline (PBS), Iba-1 (1:200; EMD Millipore, Billerica, MA, United States) was added and incubated overnight. Then, goat anti-rabbit IgG (diluted 1:200) was used to incubate the brain sections at room temperature for 10 min after washing with PBS. Subsequently, the sections were incubated at room temperature for 5 min using the DAPI dye solution (Kaiji Biological, Nanjing, China). Finally, anti-fluorescence quenching was slightly added to dry and seal the sections. Using a fluorescence microscope, the stained sections were observed, and the images were captured (×400).

### Nissl Staining and Cell Counting

In order to assess the neuronal cell death, Nissl staining was performed as previously described (Zhuang et al., 2012). The brain samples were fixed and dehydrated, paraffin-embedded, and sectioned into 10-mm thickness, followed by dewaxing for three times in xylene for 5 min each time. Then, these were placed in anhydrous ethanol for 5 min, 90% ethanol for 2 min, 70% ethanol for 2 min, and distilled water for 2 min. Afterward, the specimens were stained with Nissl staining for 10 min and rinsed twice with distilled water for a few seconds each time. Next, these were dehydrated twice in 95% ethanol for 2 min each time, and made transparent by treating with xylene twice for 5 min each time, followed by sealing with neutral gum. The specimens were viewed under a light microscope, and it was found that the neurons possessed a big cell body with a rich cytoplasm under normal conditions. In contrast, a shrunken cell body and many empty vesicles could be detected in damaged neurons. The cell count was calculated within the brain cortex. Six random high-power fields (×400) were randomly chosen to calculate the mean number of surviving neurons in each coronary section. Every third coronal section was selected, starting from 3.0 mm posterior to the optic chiasma, and four sections were collected from each animal for quantification. Finally, the average number for the 12 sections from the three individual mice brains was considered as the data for each group. The investigators presented the data as the number of surviving neurons per high-power field.

### Statistical Analysis

All data obtained in the present study was expressed as mean ± standard deviation (SD). The statistical analysis was performed by one-way ANOVA, followed by Tukey’s *post hoc* tests. *P* < 0.05 was considered statistically significant.

## Results

### Time-Course Expression and Location of S100A8 After the Experimental Traumatic Brain Injury

**FIGURE 1:**
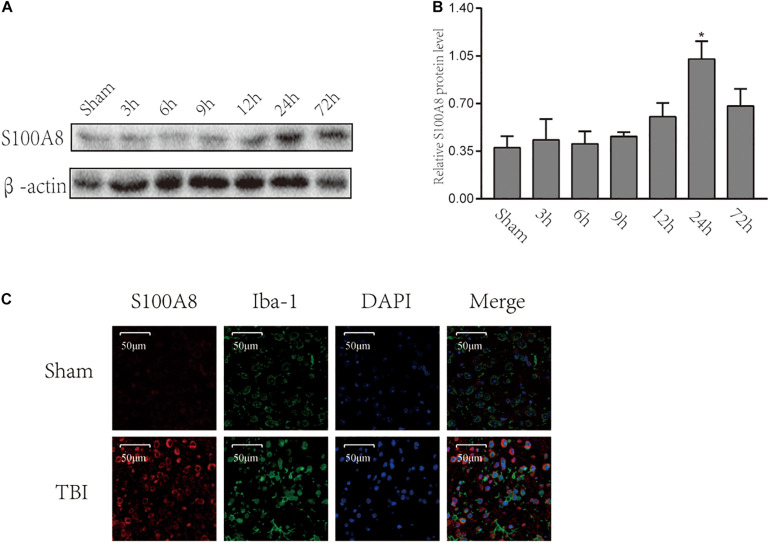
Time-course expression and location of S100A8 after the experimental TBI. **(A,B)** The time-course expression and semi-quantitative analysis of S100A8 after the experimental TBI in mice; **(C)** The representative immunofluorescence images show that S100A8 is co-localized with the microglia *in vivo*. The data was presented as mean ± standard deviation (SD; *n* = 3, per group). ^∗^*P* < 0.05, compared with the sham group, scale bars = 50 μm.

To date, there are scanty data available on the changes in S100A8 in the brain following TBI. Therefore, Western blot was performed for the brain samples obtained from the area adjacent to the contusion at different time points after the TBI (Figure 1A). The protein levels of S100A8 were not found to be significantly increased until the 24th hour after the TBI (Figure 1B). However, it was observed that the protein level of S100A8 was found to be higher at the 24th hour, when compared with its level on day 3. Therefore, the subsequent experiments were performed at the 24th-hour time point after TBI.

The immunofluorescence staining revealed the higher expression of S100A8 in the TBI group. It was detected that S100A8 was co-localized with microglia marker Iba-1 after TBI (Figure 1C). Thus, the *in vitro* study was performed in the cultured microglia BV-2.

### TLR4-MyD88-Dependent Signaling Was Activated by S100A8 or TBI

**FIGURE 2:**
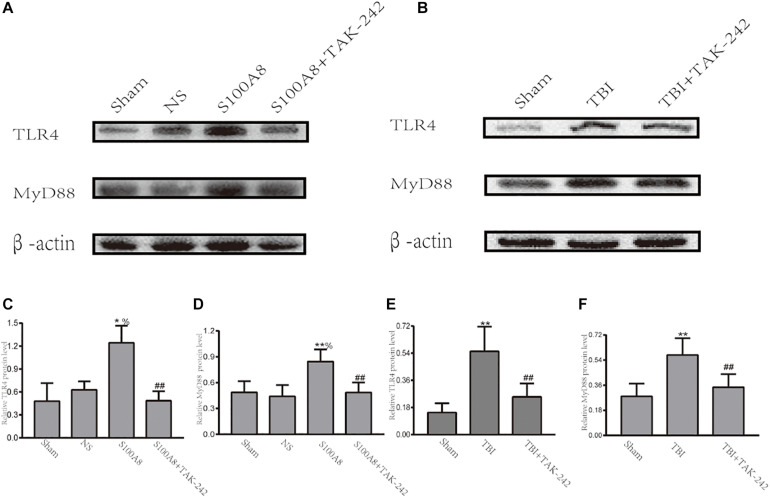
Evidence that the toll-like receptor 4 (TLR4)/MyD88 dependent signaling pathway is involved in TBI; **(A,C,D)** The representative Western blot bands and semi-quantitative analysis of the TLR4 and MyD88 expression in the different groups after the intracerebroventricular injection of S100A8 protein; **(B,E,F)** The representative Western blot bands and statistical analysis of the TLR4 and MyD88 expression in the different groups after TBI in mice. The data was presented as mean ± standard deviation (SD; *n* = 6, per group). ^∗^*P* < 0.05 and ^∗∗^*P* < 0.01, compared with the sham group; ^##^*P* < 0.01, compared with the S100A8 or TBI group. ^%^*P* < 0.05 compared to the NS group.

A number of studies revealed that TLR4 plays a significant role in initiating the inflammatory response after stroke or TBI (McCray and Taylor, 2008; Ahmad et al., 2013). Hence, it was hypothesized that S100A8 could induce the inflammation in the brain after TBI through an important inflammatory signaling pathway, that is, the TLR4-MyD88 pathway. Thus, the expression of TLR4 and MyD88 was detected in the TBI brain and the S100A8 groups. The present results revealed that the intracerebroventricular injection of S100A8 protein remarkably increased the protein levels of TLR4 and MyD88 in the brain (*P* < 0.05 and *P* < 0.01 vs. the sham group, respectively; both *P* < 0.05 vs. the NS group), in which both were lowered by TAK-242, which is an inhibitor of TLR4 (Figures 2A,C,D). The TLR4 and MyD88 levels in the TBI group were both higher, when compared to that in the sham group (both, *P* < 0.01; Figures 2B,E,F). The increase in TLR4 and MyD88 expression after TBI was partly suppressed by TAK-242 (Figures 2B,E,F).

### NF-κB Acts as the Downstream Effector of the TLR4-MyD88-Dependent Pathway After Traumatic Brain Injury

**FIGURE 3:**
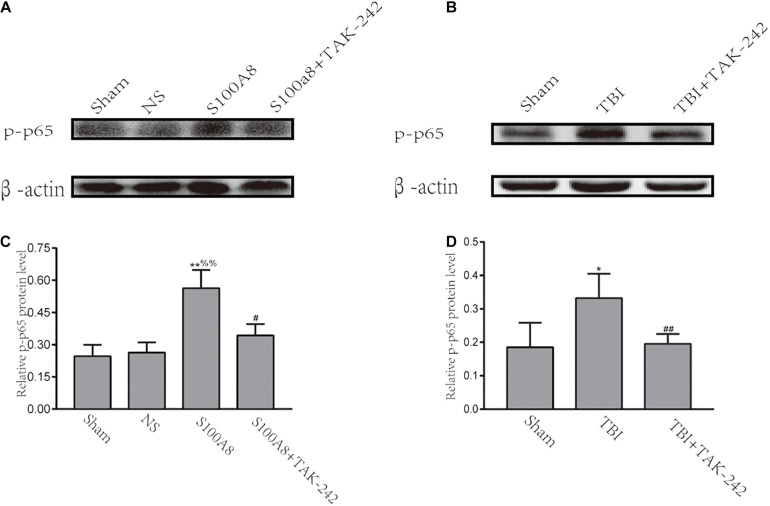
Involvement of the NF-κB activation after S100A8 stimulation and TBI. **(A,C)** The representative Western blot bands and statistical analysis of the p-p65 expression in the different groups after the intracerebroventricular injection of the S100A8 protein; **(B,D)** The representative Western blot bands and semi-quantitative analysis of the p-p65 expression in the different groups after TBI in mice. The data was presented as mean ± standard deviation (SD; *n* = 6, per group); ^∗^*P* < 0.05 and ^∗∗^*P* < 0.01 compared to the sham group; ^#^*P* < 0.05 and ^##^*P* < 0.01, when compared to the S100A8 or TBI group. ^%%^*P* < 0.01 compared to the NS group.

Previous studies have demonstrated that TLRs can trigger inflammation through the activation of NF-κB, and subsequently upregulate the pro-inflammatory cytokine expression (Vidya et al., 2017). Hence, it was revealed that NF-κB is involved in the development of inflammation in S100A8/A9-stimulated BV-2 cells (Ma et al., 2017). The investigators also hypothesized that NF-κB can act as the downstream node of the TLR4-MyD88-dependent pathway after TBI. Thus, an attempt was made to investigate the expression of phosphorylated-p65, which is the main subunit of NF-κB, at 24 h in TBI and the estimation of the S100A8 group. The results revealed that the intracerebroventricular injection of S100A8 protein or TBI markedly increased the level of p-p65, when compared with the sham group (*P* < 0.01 and *P* < 0.05, respectively), and the result of S100A8 group vs. the NS group was *P* < 0.01. In contrast, when compared with the S100A8 group or TBI group, p-p65 was found to significantly decline in both TAK-242 treatment groups (*P* < 0.01 and *P* < 0.05, respectively) (Figure 3).

### Pro-inflammatory Cytokine Production After Intracerebroventricular Injection of S100A8 Protein or TBI

**FIGURE 4:**
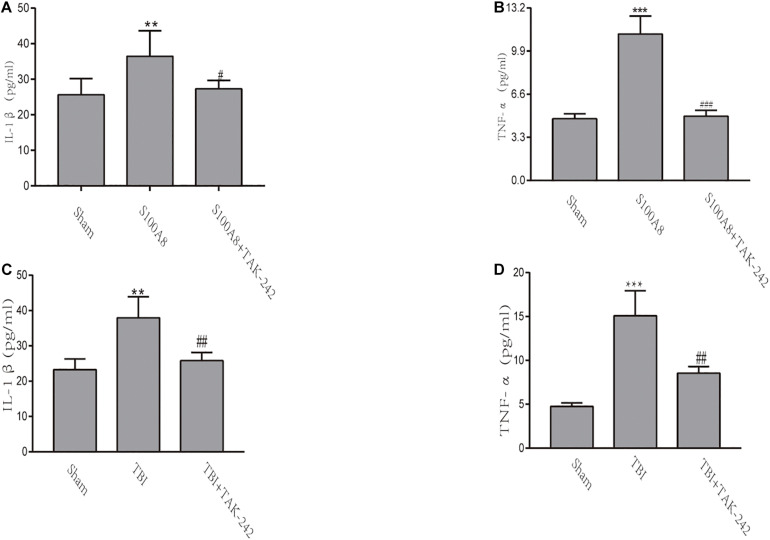
The detection of inflammatory cytokines through ELISA *in vivo*. **(A,B)** TNF-α and IL-1β levels were determined by ELISA after the intracerebroventricular injection of the S100A8 protein. **(C,D)** The pro-inflammatory cytokine production in the sham group at 24 h post-TBI, and the TAK-242 treatment of TBI mice. The data was presented as mean ± standard deviation (SD; *n* = 3, per group). ^∗∗^*P* < 0.01 and ^∗∗∗^*P* < 0.001, compared to the sham group; ^#^*P* < 0.05, ^##^*P* < 0.01 and ^###^*P* < 0.001, when compared to the S100A8 or TBI group.

The inflammatory response in TBI mice or the intracerebroventricular injection of S100A8 protein was evaluated by the levels of pro-inflammatory cytokines IL-1β and TNF-α *via* ELISA. Compared with the sham group, the concentrations of IL-1β and TNF-α significantly increased in the S100A8 group (*P* < 0.01 and *P* < 0.001, respectively) (Figures 4A,B). In contrast, the concentrations of pro-inflammatory cytokines were found to be reduced in the S100A8+TAK-242 group, when compared to the S100A8 group (*P* < 0.05 and *P* < 0.001, respectively). Although the results for the TBI and TBI+TAK-242 groups turned out to be the same as those observed in the S100A8 and S100A8+TAK-242 groups (Figures 4C,D), this suggests that S100A8 is involved in the pathology of inflammatory response after TBI.

### The Expression of p-p65, MyD88, and Pro-inflammatory Cytokines in BV-2 Microglial Cells After the Stimulation of S100A8 Protein or LPS

**FIGURE 5:**
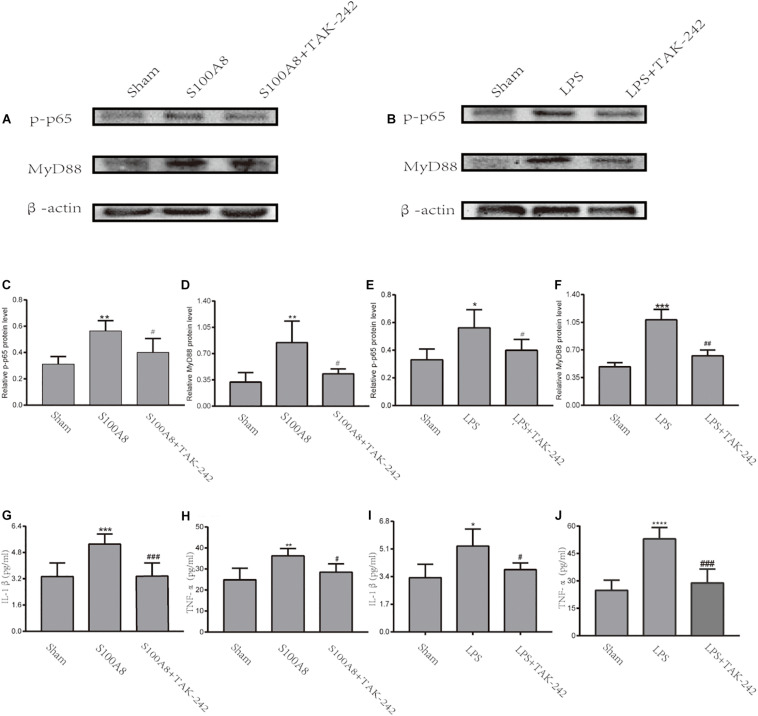
The TLR4/MyD88/NF-κB signaling pathway was activated through the stimulation of S100A8 or LPS *in vitro*. **(A,C,D)** Representative Western Blot bands and the quantitative data for MyD88 and p-p65 in the stimulation of the S100A8 protein. **(B,E,F)** The representative Western blot bands and quantitative data for MyD88 and p-p65 in the stimulation of LPS. **(G–J)** The pro-inflammatory cytokine production in BV-2 microglial cells after S100A8 or LPS stimulation. Each value represents the mean ± standard deviation (SD; *n* = 3, per group) of three independent experiments. ^∗^*P* < 0.05, ^∗∗^*P* < 0.01, ^∗∗∗^*P* < 0.001, and ^****^P < 0.0001, compared to the sham group; ^#^*P* < 0.05, ^##^*P* < 0.01, and ^###^*P* < 0.001, when compared to the S100A8 or LPS group.

To further confirm whether S100A8 enhances the production of pro-inflammatory cytokines in microglia *via* the TLR4/MyD88/NF-κB signaling pathway, the expression of MyD88 and p-p65 in the S100A8, S100A8+TAK-242, LPS, and LPS+TAK-242 groups were estimated through *in vitro* analysis. The results revealed that the treatment with S100A8 led to the remarkable increase in expression of MyD88 and p-p65, when compared to the sham group (Figures 5A,C,D) (*P* < 0.01). However, the expression of MyD88 and p-p65 in the TAK-242+S100A8 groups were found to be reduced, when compared to those in the S100A8 group (Figures 5A,C,D) (*P* < 0.05). The effects of the LPS incubation were similar to those of S100A8 (Figures 5B,E,F).

To analyze the inflammatory response in BV-2 cells stimulated by S100A8 and LPS, the expression of pro-inflammatory cytokines was detected by ELISA. As a positive control, IL-1β and TNF-α were found to be significantly elevated in the LPS group, when compared with the sham group (*P* < 0.05 and *P* < 0.0001, respectively) (Figures 5I,J). The production of IL-1β and TNF-α also dramatically increased in the cultured microglia after the stimulation of S100A8 (*P* < 0.001 and *P* < 0.01, respectively) (Figures 5G,H). At the same time, the expression of inflammatory cytokines decreased in the S100A8+TAK-242 and LPS+TAK-242 groups, when compared with the S100A8 or LPS+TAK-242 group.

Therefore, the present data suggest that the TLR4/MyD88/NF-κB signaling pathway was activated in BV-2 microglial cells incubated with S100A8 and that this subsequently promoted the production of pro-inflammatory cytokines, such as IL-1β and TNF-α.

### S100A8 and TBI Promoted Neuronal Cell Death in Mice

**FIGURE 6:**
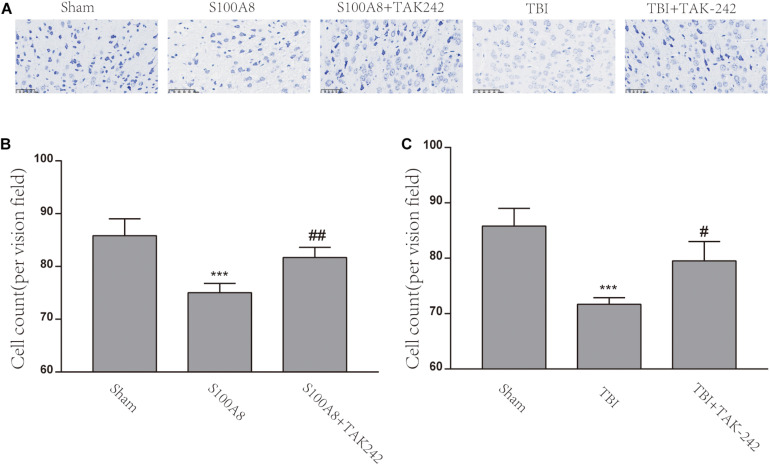
The S100A8 and TBI promoted neuronal cell death in mice. **(A)** The representative Nissl staining images of the mice brain in the different groups. **(B,C)** The statistical analysis of the cell counts of survival neurons in the different groups (*n* = 3, ^∗∗∗^*P* < 0.001, when compared to the sham group, ^#^*P* < 0.05 and ^##^*P* < 0.01, when compared to the S100A8 or TBI group).

Figure 6A explains the Nissl staining of the area adjacent to the contusion brain tissue in the different groups. The damage could be observed in the S100A8 and TBI groups, with the decrease in cell number, sparse cell arrangements, and loss of integrity. However, the treatment with TAK-242 induced an improvement in the morphological damage induced by S100A8 or TBI (Figure 6A). The number of cells also increased after the TAK-242 treatment. The quantitative results of the cell count are shown in Figures 6B,C.

### TBI and S100A8 Exaggerated the Neurological Deficits and Brain Edema, Which Were Alleviated by the TAK-242 Treatment

**FIGURE 7:**
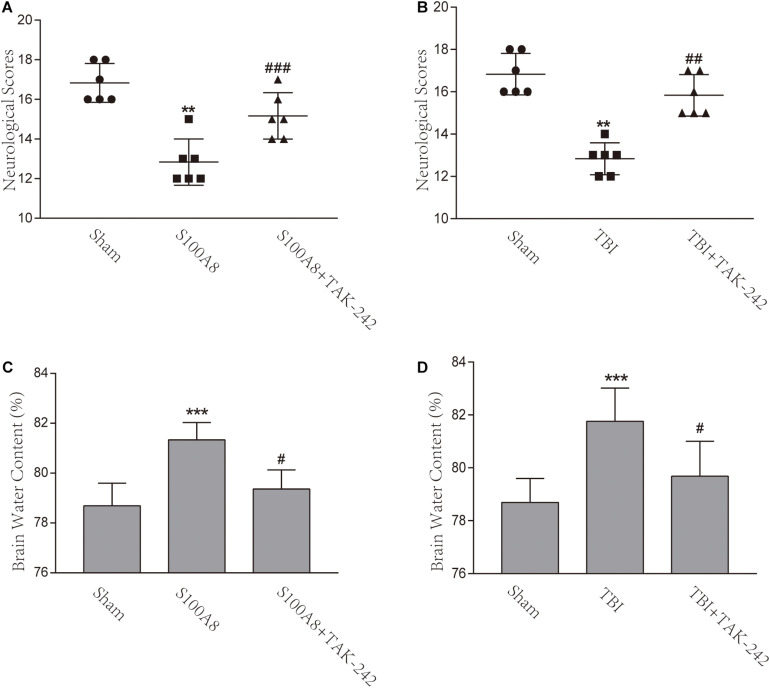
Effects of the S100A8 treatment in the neurological deficits and brain edema. **(A,B)** In the S100A8 and TBI groups, the neurological deficits were lower than those in the S100A8+TAK-242 and TBI+TAK-242 groups. The neurological scores in the sham group were higher than that in any of the other groups; **(C,D)** Brain edema was exacerbated in the TBI and S100A8 groups. The data was represented as mean ± standard deviation (SD; *n* = 6, per group). ^∗∗^*P* < 0.01 and ^∗∗∗^*P* < 0.001, when compared to the sham group; ^#^*P* < 0.05, ^##^*P* < 0.01, and ^###^*P* < 0.001, when compared to the S100A8 or TBI group.

In order to assess the neurological deficits after TBI and the intracerebroventricular injection of S100A8, the modified Garcia method was implemented in the present study. The scores gained almost full marks in the Sham group, but there was a remarkable difference found between the TBI and sham groups, as well as between the S100A8 and sham groups (*P* < 0.001, each). In addition, mice in the TBI+TAK-242 or S100A8+TAK-242 group exhibited significantly higher neurological scores, when compared with those found in the TBI group or S100A8 group (*P* < 0.01 and *P* < 0.001, respectively) (Figures 7A,B), suggesting that S100A8 is mostly harmful to the brain *via* TLR4 signaling.

The brain water content was also examined in the present study. In accordance with previous studies (Chen et al., 2017), the brain water content significantly increased in the TBI group, when compared to the sham group (*P* < 0.001) (Figure 7D). However, a considerable reduction was found after the administration of TAK-242 (*P* < 0.05) (Figure 7D). In the S100A8 group, the brain water content also increased, when compared to the sham group (*P* < 0.001) (Figure 7C), while this was suppressed after the TAK-242 treatment (*P* < 0.05) (Figure 7C).

## Discussion

The present study revealed the following findings: (i) The exogenous S100A8 can induce the activation of the inflammatory signaling pathway TLR4/MyD88/NF-κB, which is similar to TBI; (ii) TAK-242 is an inhibitor of TLR4, which could suppress the activation of TLR4/MyD88/NF-κB and the subsequent production of downstream inflammatory cytokines through S100A8 or TBI.

Previous studies have shown that S100A8 is involved in the pathophysiology of diverse diseases in the CNS, such as TBI and stroke (Ziegler et al., 2009; Zlokovic, 2011). The overexpression of S100A8 was demonstrated to play a critical role in modulating the inflammatory response through different ways of inducing cytokine secretion and the stimulation of leukocyte recruitment (Wang et al., 2018). In contrast, Goyette J et al. reported that S100A8 induces anti-inflammatory properties in macrophages (Goyette and Geczy, 2010). However, the S100A8 gene induction in macrophages is dependent on IL-10 and potentiated by immunosuppressive agents (Goyette and Geczy, 2010). Mishra et al. (2008) evidenced that S100A8 exhibited a >200-fold sensitivity to oxidative cross-linking, when compared to the low-density lipoprotein, which may reduce further oxidative damage. S100A8 and S100A9 can be S-nitrosylated (SNO), which suppresses the mast cell activation and inflammation in the microcirculation, and may act as a NO transporter to regulate the vessel tone in inflammatory lesions. Furthermore, studies have revealed that S100A8 may play a different role in different diseases, which depends on the proximal microenvironment, oligomeric form, post-transcriptional modification, and concentrations. Moreover, the intracellular S100A8/A9 was suggested to be a Ca^2+^ sensor, and binding to the Ca^2+^ changes its conformation and modulates the Ca^2+^-dependent signaling pathway. In addition, S100A8/A9 exerts both regulatory and protective functions in the cytosol (Wang et al., 2018). Thus, the main aim of the present study was to elucidate the expression and role of S100A8 in TBI. The present data revealed that the expression of S100A8 increased at the 24th hour after TBI, which could induce the proliferation of inflammatory cytokine production through the activation of the TLR4/MyD88/NF-κB signaling pathway.

Microglia and blood-derived macrophages rush rapidly toward the injury site. These are activated due to the CNS damage and are responsible in initiating the pathophysiological conditions. Microglia produces cytokines and trophic factors that can exert damaging or protective effects on neighboring cells. In response to a variety of ligands, TLR4, which is mainly expressed in microglia in CNS, promotes the microglial activation and expression of pro-inflammatory mediators (Mccoll et al., 2009). Furthermore, TLR4 signaling results in the activation of NF-κB, and subsequently drives the abundant transcription process of pro-inflammatory cytokines that may cause tissue destruction and the activation of the innate immune system (Karikó et al., 2004; Kielian, 2006; Mishra et al., 2008). During septic shock, S100A8 acts as an endogenous ligand of TLR4 and induces the elevated expression of TNF-α in phagocytes, which in turn induces the translocation of MyD88 and activation of NF-κB (Vogl et al., 2007). Similarly, the present data demonstrated that the protein of S100A8 may cause inflammation after TBI *via* the TLR4/MyD88-dependent pathway.

The protein of S100A8 has a strong positive effect on the inflammatory signaling pathway (i.e., NF-κB), which is in accordance with the results. NF-κB can be considered as one of the most important transcription factors characterized in the brain at present, and this might be crucial for neuronal and glial cell function. According to the findings in previous studies, NF-κB is considered as a central regulator of microglia, which responds on active stimuli (O’Neill and Kaltschmidt, 1997). In the present study, S100A8 treatment significantly enhanced the levels of p-p65 *in vitro* and *in vivo*. Hence, the involvement of NF-κB in the induction of inflammatory response through S100A8 after TBI was confirmed.

The present data revealed that S100A8 represents a molecular system that participates in the pathogenesis of the inflammatory response upstream of the TNF-α and IL-1β induction. Due to the high abundance of S100A8 and its involvement in inflammatory diseases, a potential therapy for inhibiting the uncontrolled inflammatory process by targeting is feasible.

In the present study, experiments had limitations, which need to be resolved. It remains unclear whether the augment of S100A8 is prior to the increased inflammatory cytokines after TBI. Therefore, the detection of cytokine production in different time courses after TBI is necessary.

## Conclusion

In summary, the present data indicates that S100A8 promotes the production of pro-inflammatory cytokines *via* the activation of the TLR4/MyD88/NF-κB signaling pathway after TBI. Thus, S100A8 can be considered and serves as a potential biomarker for inflammation in the brain after TBI. Furthermore, the inhibition of S100A8 may partly alleviate the adverse consequence after TBI, and which could be a target for providing the therapy to TBI. In future studies, the investigators will focus on modulating the TLR4/MyD88/NF-κB signaling cascade that could limit the activation of pro-inflammatory cytokines, and this might eventually reduce the TBI susceptibility.

## Data Availability Statement

The raw data supporting the conclusions of this article will be made available by the authors, without undue reservation.

## Ethics Statement

The animal study was reviewed and approved by the Animal Care and Use Committee of Jinling Hospital.

## Author Contributions

G-YH and C-HZ contributed in the design, conception, and writing of the manuscript. D-GW and HC participated in several experiments and carried out the analysis, acquisition, and interpretation of the data. L-AS, D-LZ, X-JY, and X-RF made the statistical analysis and revision of the manuscript. G-FD and X-CJ supported in the technical support, such as conception, design, funding, and revision of the manuscript. All authors contributed to the article and approved the submitted version.

## Conflict of Interest

The authors declare that the research was conducted in the absence of any commercial or financial relationships that could be construed as a potential conflict of interest.
